# Infantile Convulsions

**Published:** 1868-03

**Authors:** John Dickson


### INFANTILE CONVULSIONS.

By JOHN DICKSON, M.D.

Read before the Baltimore Medical Association.

Of all the maladies of infancy, I know of none more serious, or embarrassing to treat, than convulsions. We are summoned in great haste, and arrive out of breath, and find a painful scene of dismay and confusion which requires our utmost tact and composure to lull. We never get *used* to spasms in the sense in which we may become stoically or philosophically calm. The malady is too grave and sudden in its effects, and our responsibility too serious for that. We all know that two or three minutes of tonic spasm, or a few hours of clonic, may destroy life, and how much depends upon the prompt and judicious action of the physician, both for the child's safety and his own reputation. Unless the case occurs in our own family, or has been attended with premonitory symptoms, we rarely see a spasm in the tonic stage. The violent and sustained contraction of the respiratory muscle stops the breathing, and death results in a few minutes from asphyxia, as in the first stage of epileptic fits, or in fatal cases of laryngismus stridulus. This, fortunately, is a rare occurrence. We generally find the state, which soon succeeds, of alternate retraction and relaxation, either general or partial; and sometimes confined to very few muscles, as those of the face or hand. In severe cases there is violent jerking of the limbs, abduction of the thumbs covered by the contracted fingers, staring or rolling, insensible eyes, with pupils either contracted or dilated, or there is strabismus in the course of the spasm; the head is drawn backward or forward from the beginning, or it is twisted in rotatory movements; the respiration is quick and irregular, producing a sound of choking as distressing to hear as the contracted, livid face is to witness. When clonic spasm continues some hours, and the rapid contraction and relaxation prevent the free egress of the carbonized air from within the lungs, or the admission of enough pure air to renew the blood, both circulation and respiration are arrested, and death must follow.

Even when this is not the immediate result of the spasm, in many instances it occurs within a short time after it subsides, because these vital functions have been so seriously impaired as to preclude respiration, and the patient sinks from the shock—as we have heard mothers expressly say, "they were struck with death from the beginning of the attack."

But, happily, this is not the most common course of convulsions. There is oftener a gradual subsidence of the fit. The spasmodic movements become slower and cease, the respiration is free, and a general calm succeeds. The patient either wakes to consciousness, or falls into a comotose, or, it may be, natural sleep, after which there may or may not be a recurrence of the spasms. Such recurrences are very common, in spite of our best efforts to prevent them, but it is gratifying to know that the danger is not proportionally increased by their frequency. I have seen children have six or seven in the course of the day, and on the following day present no unfavorable symptom or spasm ever afterwards.

Partial spasms present such a variety of forms that I shall not attempt to speak of more than one or two. They may be confined to the superficial muscles only, and to a few of these, and in such cases, the senses remain intact. I have seen the eye, mouth, and hand of one side jerking, while the sensibility of the child was perfect; and it would ask for milk and drink it, attempting to hold the cup with the convulsed hand and steadying it with the other. Such are the sequelæ of the more severe attacks, and are easily excited in children so predisposed. Sometimes the muscles of the neck are alone effected, and cause rotation or flexion of the head forward or backward. Indeed, single muscles, as well as sets of muscles, in almost every part of the body, may be convulsed, or exhibit movements under peculiar excitement, nervous or fibrile, which closely resemble spasm, and are at times mistaken for such. Certain organs alone may be effected, as the larynx or glottis, and we have a very formidable trouble in laryngismus stridulus. A child may be seized without any premonitory symptoms of dangerous import, with apparent suffocation. His breathing is suspended, his head thrown back, face and lips livid, and, in a few seconds, the spasm yielding, respiration follows, and a sudden gasp for breath, so urgent as to produce a crowing sound; and the breathing goes on naturally. But there are cases so violent from the repetition of these spasms, as to destroy life during the paroxysm, or lead to general convulsions and coma. Marshall Hall calls this affection "an excitation of the true spinal or excito-motory system." It originates in the trifacial in teething; in the pneumogastric in over or improperly fed infants; in the spinal nerves in constipation, intestinal disorder or catharsis. These act through the medium of the spinal marrow, and the inferior or recurrent laryngeal, the constrictor of the larynx, and the intercostals and diaphragmatic, the motors of respiration." We can judge from this that a great variety of causes, as in general convulsions, may produce this form of spasm. Dr. West mentions a case in a child only ten weeks old, from improper feeding; another, of nineteen months, from sudden suppression of chronic diarrhœa; another, of two and a-half years, from cerebral congestion following constipation; another, of nine months, during the course of chronic hydrocephalus; and in another, who died at the age of two months, it appeared as a transitory symptom during a series of convulsive attacks, for which no cause could be assigned during life, and which left no traces that could be detected after death.

The obscurity of origin and absence of pathological indications often throw a veil of mystery over cases of convulsion, which the clearest sighted of us cannot penetrate. We know that hereditary influence is the most frequent predisposing cause, that eclampsia in the mother before parturition, or much further back, when she was herself a child, is apt to be followed by the same tendency in the offspring; though it by no means follows so often as to establish it as a rule.

A remarkable illustration of hereditary influence is quoted by Trousseau from a thesis of Dr. Duclos, of Tours. The case is that of a woman, thirty-four years of age, who had had frequent attacks of eclampsia up to the age of seven. These had left behind slight deviation of the mouth and ptosis of the left upper eyelid. This woman had ten children, who all had convulsions; six had died, five in the first two years, and one when three years old. Three months previously, she had a first attack, which lasted about ten minutes, and which her mother ascribed to her having given the breast to the child immediately after a fit of passion, as the convulsion occurred on the ensuing day. Death took place three months afterward, from cerebro-meningitis.

Loss of blood, whether in direct hæmorrhage, venesection, diarrhœa, or hypercatharsis, strongly predisposes to convulsions. Insufficient nourishment and exhaustion, from whatever cause, have the same effect. Hippocrates' observation that the "blood is the moderator of the nerves," corresponds with the present physiological law, "that in proportion as the nutritive and vegetative functions are feeble and languishing, nervous phenomena are mobile, exalted and irregular." The sensitive brain, with its spirit-like nerves prevading every part of the organism, must be supported by the vascular system, as the string and wind instruments of an orchestra combine; the measured wave sounds of the latter, giving volume and tone to the tender strains of the former, without which it would be only a flutter of distracting discord. The iron of the blood is as much a fundamental base in toning the system, as the brass instruments are in sustained musical harmony. It may be upon this theory that Chapman uses ice bags along the spine in so many affections where nervous symptoms predominate. "He considers that ice applied along the spine increases the general circulation, stops the cramp of voluntary and involuntary muscles, proves an effective remedy in epilepsy and other convulsive affections, cures sea-sickness, restrains the sickness of pregnancy, arrests diarrhœa, recovers patients from the cold stage of cholera, and, finally, promotes menstruation. On the other hand, heat along the spine lessens the general circulation, overcomes congestion in all parts of the body, lessens fever, restrains hæmorrhage and lessens or arrests the menstrual flow." If by exciting or depressing the spinal cord, by heat or cold, such remarkable effects can be produced upon the circulatory system, we can readily see how disorders of the latter may prove disastrous to the nervous system. To work out this would be very interesting, but would require more time than I can give it. The fact is, that every thing that impedes or arrests healthy circulation, or impairs the quality or quantity of the blood, may tend to bring on convulsions, and may be ranked among the predisposing causes. The exciting causes of convulsions are very numerous, and upon them, when we can discover them, we base our immediate treatment of an attack. When arising from indigestion, how often have we cut short the spasm by an emetic or enema, when nature herself has not done the work for us, which she frequently does, and when constipation is the cause by relaxing the sphincter and evacuating the loaded intestines. But these measures often fail, and in spite of the inevitable warm bath and counter-irritation, which the child's friends have applied before our arrival, the convulsion goes on unabated; and if we do not arrest it, the child may die in the fit or from the supervening coma. Almost all the antispasmodics have been used for this purpose, and some of them with good effect at times, but no agent is so powerful or requires more skill in administering than chloroform. Some bear it very badly, and we discover the flagging of the pulse or the stertor of the breathing very soon after its application, and we must desist before any good can be done by it. In other cases its use may be kept up for a long time with no bad effects, and the convulsive action controlled. In severe cases I have seen the chloroform used freely for several hours, and the child recovered perfectly, when without it the paroxysm would have undoubtedly exhausted the nervous system, or produced cerebro-meningitis, or effusion and resulting paralysis. Such are often the results of convulsions, besides deformities from rupture of muscles, squinting, nervous excitability, epilepsy, etc., though by no means occurring in a large proportion of cases. In some children convulsions are easily excited and readily controlled, and the agent which I have found most valuable for this purpose is bromide of potassium. There is still a good deal of skepticism on this point, but I think where it has been used and persisted in, there is no doubt of its efficacy in preventing and subduing nervous excitability. I have given it, when the convulsive tendency was the result of impoverished blood from previous disease, in conjunction with wine and beef essence, with the happiest effect.

Of the effect of ice to the spine I have no testimony of my own. Dr. Edmunds, in the *Medical Times* of March 12th, 1864, after relating a case of spasm in a woman which was perfectly relieved by this means, says: "I had seen Dr. Chapman's brochure on the subject of his discovery, and also his paper in the *Medical Times and Gazette*, but thought the idea too pretty to be anything more than a plausible theory, until my own child, being in great danger from an obstinate laryngismus, connected with dentition, I tried the ice bag to the cervico-dorsal portion of the spine, at the suggestion of Dr. Ramskill, and it has certainly done more to keep off the strangling attacks than anything else.

Lancing the gums when the convulsions occur during dentition, sometimes produces immediate relief, and, if they occur during that period, it should be our first duty.

Convulsions which take place at the outset of fevers, from the first impression of the poison upon the system, are not as serious as those which come on towards the close, and seldom require special treatment. It is a disputed point whether the prognosis from such is favorable or not. Worms, sunstroke, extremes of temperature, blows upon the head, sudden fright, severe burns, and local irritation of various kinds, are among the exciting causes of convulsions, and indicate the course of treatment. Calomel has been given very largely in convulsions, as a purgative, anthelmintic and absorbent. We must bear in mind its destructive tendency, and have a distinct purpose in view, the accomplishment of which is paramount to the risk of using such a depleting agent. If we give it to promote absorption of effused serum in the brain we may obviate its injurious effects and increase its efficiency by taking especial pains to nourish and support the general system at the same time, with all the means the patient will bear.

This plan was successful in my own little girl, who was two and a-half years old when she was attacked with a violent convulsion, after a day or two of gastric irritation, from which she seemed to be recovering, when, without any warning, she was seized with it, and, in spite of chloroform and everything else we could do for her, it lasted five and a-half hours. The right side was most effected, and, after the convulsion, remained paralyzed for several days, but recovered under the use of small doses of calomel combined with bromide of potassium, and wine, milk, and beef essence. There was a convulsive state for some weeks afterward, which seemed to be controlled by the bromide, and partial convulsions occurred without loss of consciousness, for some days after her recovery from the comatose condition which immediately followed the convulsion. The paralysis left the foot first, and she could walk well for some days before she could hold anything in her hand, but that gradually regained its use, and lastly, her tongue, which had remained silent for four weeks, began to liberate itself, in monosyllables at first, and she slowly recovered her vocabulary, as if she had never talked before. This case, which I had hoped to give more in detail, was one of intense interest and anxiety to me, and the care and responsibility of the treatment was most kindly and faithfully shared by our worthy president (Dr. Williams). She is held up in our neighborhood as a trial of medical skill, and an encouragement to parents, as well as doctors, to hope for the recovery of their little ones under the most discouraging circumstances.

